# The cost-effectiveness of diabetes prevention: results from the Diabetes Prevention Program and the Diabetes Prevention Program Outcomes Study

**DOI:** 10.1186/s40842-015-0009-1

**Published:** 2015-09-02

**Authors:** William H. Herman

**Affiliations:** Diabetes Prevention Program Coordinating Center, The Biostatistics Center, George Washington University, 16110 Executive Blvd., Suite 750, Rockville, MD 20852 USA

**Keywords:** Cost, Quality-of-life, Cost-utility

## Abstract

**Background:**

The Diabetes Prevention Program (DPP) was a randomized, controlled clinical trial. It demonstrated that among high-risk individuals with impaired glucose tolerance, diabetes incidence was reduced by 58 % with lifestyle intervention and 31 % with metformin compared to placebo. During the Diabetes Prevention Program Outcomes Study (DPPOS), all DPP participants were unmasked to their treatment assignments, the original lifestyle intervention group was offered additional lifestyle support, the metformin group continued metformin, and all three groups were offered a group-implemented lifestyle intervention. Over the 10 years of combined DPP/DPPOS follow-up, diabetes incidence was reduced by 34 % in the lifestyle group and 18 % in the metformin group compared to placebo. The purpose of this article is to review and synthesize analyses published by the DPP/DPPOS Research Group that have described the cost-effectiveness of diabetes prevention.

**Methods:**

We describe the resource utilization and costs of the DPP and DPPOS interventions, the costs of non-intervention-related medical care, the impact of the interventions on diabetes progression and quality-of-life, and the cost-effectiveness of the interventions from health system and societal perspectives. Cost-effectiveness analyses were performed with a 3-year time horizon using DPP data, a lifetime time horizon that simulated 3-year DPP data, and a 10-year time horizon using combined DPP/DPPOS data.

**Results:**

Although more expensive than the placebo intervention, the greater costs of the lifestyle and metformin interventions were offset by reductions in the costs of nonintervention-related medical care. Every year after randomization, quality-of-life was better for participants in the lifestyle intervention compared to those in the metformin or placebo intervention. In both the simulated lifetime analysis and the 10-year within trial economic analysis, lifestyle and metformin were extremely cost-effective (that is, improved outcomes at a low incremental cost) or even cost-saving (that is, improved outcomes and reduced total costs) compared to the placebo intervention.

**Conclusions:**

The implementation of diabetes prevention programs in high-risk individuals will result in important health benefits and represents a good value for money.

**Trial registration:**

NCT00004992 (DPP) and NCT00038727 (DPPOS).

**Electronic supplementary material:**

The online version of this article (doi:10.1186/s40842-015-0009-1) contains supplementary material, which is available to authorized users.

## Introduction

The Diabetes Prevention Program (DPP) was a multicenter clinical trial designed to determine whether modest weight loss through dietary changes and increased physical activity or treatment with the oral antihyperglycemic medication metformin could delay or prevent the development of type 2 diabetes in high-risk individuals [1]. The DPP enrolled 3234 participants with glucose intolerance who were at least 25 years of age and had a body mass index of 24 kg/m^2^ or higher (22 kg/m^2^ in Asian-Americans). Mean age of participants was 51 years and mean BMI was 34.0 kg/m^2^. Sixty-eight percent of participants were women and 45 % were members of minority groups.

The goals for participants randomized to the intensive lifestyle intervention were to achieve and maintain a weight reduction of at least 7% of initial body weight through a low-calorie, low-fat diet and physical activity of moderate intensity, such as brisk walking for at least 150 min per week [2]. A 16-lesson core curriculum addressing diet, physical activity, and behavior modification was implemented to help participants achieve these goals. The curriculum, taught by case managers on a one-to-one basis during the first 24 weeks after enrollment, was flexible, culturally sensitive, and individualized. Subsequent individual sessions (usually monthly) and group sessions were designed to reinforce the behavioral changes.

The medication interventions (metformin and placebo) were initiated at a dose of 850 mg taken orally once a day [3]. At one month, the dose of metformin or placebo was increased to 850 mg twice daily unless gastrointestinal symptoms warranted a longer titration period. Participants were seen by case managers and adherence to the treatment regimen was reinforced quarterly. The standard lifestyle recommendations for the medication groups were provided in an annual 20 to 30 min individual session. The DPP demonstrated that compared to the placebo intervention, the intensive lifestyle intervention reduced the incidence of type 2 diabetes by 58 %, and the metformin intervention reduced the incidence of type 2 diabetes by 31 % over 3 years [1].

At the conclusion of DPP, participants were enrolled in the Diabetes Prevention Program Outcomes Study (DPPOS). DPPOS was designed to assess the long-term effects of the interventions on health [4]. During DPPOS, participants originally randomized to the lifestyle and metformin interventions were encouraged to continue those interventions and all participants were offered a group lifestyle intervention. The incidence of diabetes during the 10-year average follow-up after DPP randomization was reduced by 34 % in those initially randomized to lifestyle and 18 % in those initially randomized to metformin compared to placebo [4].

To date, we have reported the resources used and the costs of care, and the cost-effectiveness of the lifestyle and metformin interventions relative to the placebo intervention over the 3 year timeframe of the randomized controlled clinical trial [5, 6]. We also used data collected during the 3 years of the DPP and a computer model to simulate the cost-effectiveness of the interventions over a lifetime [7]. Although we [7] and others [8, 9] suggested that the DPP interventions would be cost-effective or even cost-saving over the long term, one analysis suggested that they might be too expensive to be routinely implemented [10]. To better address the longer-term cost-effectiveness of the DPP interventions, we subsequently performed a within-trial analysis spanning the combined 10-years of DPP/DPPOS [11]. In this report, we synthesize and discuss the results of these published reports.

## Review

### Methods

We described the direct medical costs, direct non-medical costs, and indirect costs incurred by participants in the lifestyle, metformin, and placebo intervention groups during DPP [5] and DPPOS [11]. In general, we calculated the direct medical costs of the interventions by assessing resources used and applying standard unit costs [5]. We excluded from the analysis the resources used and costs of developing the interventions and collecting outcomes to evaluate the interventions [5]. The direct costs of medical care received outside the study and indirect costs were determined annually from patient self-report. Direct non-medical costs were assessed once during DPP and once during DPPOS, and costs were annualized. All costs were adjusted to 2000 or 2010 U.S. dollars using the Consumer Price Index and the Medical Consumer Price Index.

In our analyses, we adopted two separate perspectives: the perspective of a large health system and the perspective of society. In the analyses that adopted the perspective of a health system, we considered the direct medical costs of the DPP/DPPOS interventions and the direct medical costs of care received outside the study. In the analyses adopting the perspective of society, we considered direct medical costs, direct non-medical costs, and indirect costs.

Direct medical costs represent expenditures for medical services and products that are usually paid by health systems. These costs include the costs of hospital days, emergency room visits, urgent care (immediate care for injuries and illnesses in a medical facility outside of a traditional emergency room) visits, outpatient visits, calls to providers, supplies, laboratory tests, and prescription medications. In estimating direct medical costs, we considered the costs of the interventions and the costs of non-intervention-related medical care received outside the DPP/DPPOS. Direct non-medical costs represent expenditures arising as a result of medical treatment or illness but not involving the purchase of medical services or products. Since these costs do not represent health care expenditures, they are not usually paid by health systems. They do, however, represent “out-of-pocket” costs to patients and costs to society. In DPP/DPPOS, direct non-medical costs included the value of the time that participants spent traveling to and attending appointments, exercising, shopping, and cooking; the costs of exercise classes, exercise equipment, special foods, and food preparation items; and the costs of transportation to and from appointments. Indirect costs are another cost to society that arise from illness-related morbidity and mortality. Indirect costs from morbidity arise from being absent from work due to medical treatment, illness, or long-term disability. Indirect mortality costs arise from lost productivity due to premature death.

We performed cost-utility analyses by comparing costs to outcomes across intervention groups. When an intervention costs less and improves outcomes relative to an alternative treatment, it is called cost-saving. When an intervention costs more but improves outcomes relative to an alternative treatment at a cost per unit outcome considered to represent a good value for the money spent, it is termed cost-effective. Outcomes were expressed in terms of quality-adjusted life-years (QALYs) [6]. QALYs measure length of life adjusted for quality of life. Mathematically, QALYs are calculated as the sum of the product of the number of years of life and the quality-of-life in each of those years. The numerical value assigned to quality of life is called a health utility score. By convention, health utility scores are placed on a continuum where perfect health is assigned a value of 1.0 and health judged equivalent to death is assigned a value of 0.0. We assessed health utility scores from the perspective of the general public using the Self-Administered Quality of Well-Being Index (QWB-SA) which was administered to DPP participants annually.

At the end of DPP, we performed a 3-year within trial economic analysis of the DPP and used a simulation model to estimate the lifetime cost-effectiveness of the interventions. The simulation model was originally developed by the Centers for Disease Control and Prevention and Research Triangle Institute International to assess the progression from impaired glucose tolerance to the onset of diabetes to clinically diagnosed diabetes to diabetes with complications and comorbidities to death [12]. The model has a Markov structure and includes annual transition probabilities between disease states. In addition to disease progression, the model tracks costs and QALYs. For our analyses, we modified the model to include data from the DPP on progression, costs, and quality of life associated with impaired glucose tolerance; data from the United Kingdom Prospective Diabetes Study (UKPDS) on diabetes progression, complications, and comorbidities; and new data on the cost and quality of life associated with diabetes. To estimate the costs of type 2 diabetes, we applied a multiplicative prediction model that estimates annual direct medical costs according to demographic characteristics, diabetes treatments, cardiovascular risk factors, and microvascular and macrovascular complications and comorbidities [13]. To estimate health utility scores associated with type 2 diabetes, we applied an additive prediction model that estimates health utility scores according to demographic characteristics, treatments, and disease state variables [14]. We then modeled the interventions as they were implemented in the DPP and projected year 3 DPP intervention costs, health utility scores, and intervention effectiveness into the future. We assessed simulated lifetime costs and QALYs and calculated cost-effectiveness ratios by dividing incremental costs adjusted to year 2000 U.S. dollars by incremental QALYs. We discounted both costs and QALYs at 3 % per year.

In sensitivity analyses, we assessed how robust the results of the lifetime simulation were to plausible changes in the inputs. First, we modeled the interventions by age group. Then, we modeled the interventions as they might be implemented in routine clinical practice and assessed the effect of reducing the costs of the interventions. Specifically, we recalculated the cost of the lifestyle intervention, assuming that the core curriculum, supervised activity sessions, and lifestyle group sessions were administered as a closed group of 10 participants and that costs were reduced accordingly. Similarly, we recalculated the cost of the metformin intervention by using the cost of generic metformin priced at 25 % the cost of Glucophage (Bristol-Myers Squibb, Princeton, New Jersey). Then, we evaluated the impact of reduced participant adherence by reducing the effectiveness of the lifestyle and metformin interventions by 20 % and 50 % after year 3. Finally, we evaluated the impact of both reduced costs and reduced effectiveness on lifetime cost-effectiveness.

In the 10-year within trial economic analysis, we calculated the total direct medical costs and QALYs for participants over 10 years according to their original DPP randomization groups and assessed cost-effectiveness without simulation modeling but using the empiric data [11]. As a sensitivity analysis, we again estimated what the cost of the lifestyle intervention might have been if it had been administered during DPP in a group format rather than individually (DPP group lifestyle intervention). Although metformin was implemented with brand name metformin (Glucophage), we again assumed that it was implemented with generically-priced DPPOS/DPPOS.

## Results

### Direct medical costs of the DPP/DPPOS interventions

During both DPP and DPPOS, the lifestyle and metformin interventions were substantially more expensive than the placebo intervention. Table 1 shows the undiscounted per capita direct medical costs of the DPP/DPPOS interventions by intervention group and study year [5]. The costs of the lifestyle intervention were less during DPPOS than during DPP because of the change from an individual- to a group- implemented intervention and because fewer visits took place [11]. The costs of the placebo intervention were higher during DPPOS than during DPP because placebo participants engaged in the group lifestyle intervention [11]. The cumulative undiscounted per participant cost of the lifestyle intervention ($4572) was substantially greater than the metformin intervention ($2881) or the placebo intervention ($752). The estimated cost of the DPP group lifestyle intervention ($2995) was approximately one-third less than that of the lifestyle intervention.

**Table 1:** Undiscounted per capita direct medical costs of the DPP/DPPOS interventions by intervention group and study year ($)

Year	Lifestyle	Metformin	Placebo	DPP Group Lifestyle^a^
1-DPP	1,826	584	87	898
2-DPP	887	294	50	563
3-DPP	915	299	47	590
4 (Bridge)	173	301	220	173
5-DPPOS	126	138	62	126
6-DPPOS	112	136	61	112
7-DPPOS	139	137	59	139
8-DPPOS	138	132	55	138
9-DPPOS	126	131	55	126
10-DPPOS	130	130	55	130
Total	4,572	2,281	752	2,995

^a^Sensitivity analysis. Assumes that the core curriculum and follow-up visits were conducted as group sessions with ten participants during the 3 years of DPP

### Direct medical costs of care received outside the DPP/DPPOS interventions

To estimate the costs of medical care outside the DPP/DPPOS, we assessed the mean per capita cost of hospital days, emergency room visits, urgent care visits, outpatient visits, calls to providers, supplies, laboratory tests, and prescription medications within the intervention groups [5]. Table 2 shows the undiscounted per capita direct medical costs of care outside the DPP/DPPOS. Cumulative per capita direct medical costs of care outside the DPP/DPPOS were least for the lifestyle group ($24,563), intermediate for the metformin group ($25,615), and highest for the placebo group ($27,468) indicating that metformin and lifestyle participants used fewer medical resources outside the DPP/DPPOS interventions than participants randomized to the placebo intervention [5]. The cumulative per-participant direct medical costs of non-intervention-related medical care increased substantially over time. The direct medical costs of non-intervention related medical care were substantially greater than the costs of the interventions and by 3 years, the cumulative costs of non-intervention-related medical care exceeded the 10-year cumulative direct medical costs of the interventions [11]. The greater cost of non-intervention-related medical care for the placebo group was largely driven by greater use of outpatient and inpatient services, prescription medications, and by the greater rate of conversion to diabetes with the attendant costs of self-monitoring and laboratory tests (Table 2). Across treatment groups, the direct medical costs of non-intervention-related medical care were 34 to 44 % higher among diabetic participants compared to nondiabetic participants.

**Table 2:** Undiscounted per capita direct medical costs of care outside the DPP/DPPOS by intervention group and study year, and distribution of undiscounted per capita 10-year direct medical costs of care outside the DPP/DPPOS by intervention group and type ($)

Costs by year	Lifestyle	Metformin	Placebo
1-DPP	1,423	1,517	1,617
2-DPP	1,780	1,837	2,045
3-DPP	1,979	1,854	2,018
4 (Bridge)	2,059	2,087	2,330
5-DPPOS	2,015	2,174	2,543
6-DPPOS	2,519	2,493	2,636
7-DPPOS	2,645	3,061	2,875
8-DPPOS	3,444	3,607	3,319
9-DPPOS	3,291	3,298	3,265
10-DPPOS	3,406	3,686	4,822
TOTAL	24,563	25,615	27,468
Costs by category	Lifestyle	Metformin	Placebo
Outpatient visits	6,845	7,145	7,325
Inpatient care	5,631	5,817	6,856
ER visits	1,941	1,690	1,825
Urgent care visits	1,697	1,945	1,811
Calls to physicians	712	742	712
Prescription medications	6,490	6,619	6,959
Self-monitoring supplies and laboratory tests for diabetes	1,248	1,628	1,978
TOTAL	24,563	25,615	27,468

### Total direct medical costs

By year 10, the cumulative undiscounted, per capita, total direct medical costs of the interventions and non-intervention-related medical care were higher for lifestyle ($29,135) than for placebo ($28,040) but were lower for metformin ($27,896) than for placebo ($28,040). They were also lower for the DPP group lifestyle ($27,558).

### Direct Non-medical costs

Participants randomized to the three intervention groups reported that they spent different amounts of time attending appointments and traveling to and from appointments, exercising, shopping, and cooking and that they received different levels of enjoyment from leisure-time physical activity [5, 11]. They also reported different out-of-pocket costs related to purchases of services and products related to physical activity and diet, different expenditures for food, and different transportation costs [5, 11].

Diet-related costs were substantial but did not differ among intervention groups. As might be expected, physical activity-related costs were greatest for lifestyle. Transportation-related costs were also substantially higher for lifestyle and metformin, due to the greater number of study visits. Total diet-related, physical activity-related, and transportation-related costs were greatest for lifestyle but similar for metformin and placebo.

Participant time contributed substantially to direct nonmedical costs. Participant time related to the interventions (time spent traveling to study visits, at study visits, and for intervention-related calls) was greater for lifestyle and metformin than for the placebo. Participant time related to medical care outside of the interventions was generally greater for placebo than for metformin or lifestyle. Time spent shopping and cooking was the largest component of participant time but differed little across intervention groups. Although lifestyle subjects spent more time exercising, the adjusted value of the time they spent exercising was less than for either metformin or placebo because of their greater enjoyment of leisure time physical activity and the lower opportunity cost.

The total per capita 10-year direct nonmedical costs including the costs of participant time were lowest for metformin ($144,143) and similar for placebo and lifestyle ($147,043 and $147,493 respectively) [11].

### Indirect costs

Participants in the three intervention groups reported small differences in time lost from school, work, or usual activities as a result of study visits, illness, or injury. In general, participants in the placebo and metformin groups reported more time lost than participants in the lifestyle group [11].

### Health utility scores

Every year after randomization, quality-of-life was better for participants in the lifestyle intervention than for those in the metformin or placebo interventions [11]. Across treatment groups, quality-of-life was worse among participants who developed diabetes [11]. Since more placebo participants developed diabetes, the cumulative, undiscounted, per-participant QALYs-gained over 10 years was greatest for lifestyle (6.81), intermediate for metformin (6.69), and least for placebo (6.67) [11].

### Within DPP 3-year cost-effectiveness analysis

From the perspective of a health system and compared to the placebo intervention, the lifestyle intervention cost $31,500 per QALY-gained and the metformin intervention cost $99,600 per QALY-gained [6]. From the perspective of society and compared to the placebo intervention, the lifestyle intervention cost $51,600 per QALY-gained and the metformin intervention cost $99,200 per QALY-gained [6]. The lifestyle intervention was more cost-effective than the metformin intervention from the perspective of both a health system and society.

### Simulated lifetime cost-effectiveness of the DPP interventions

**Fig. 1:**
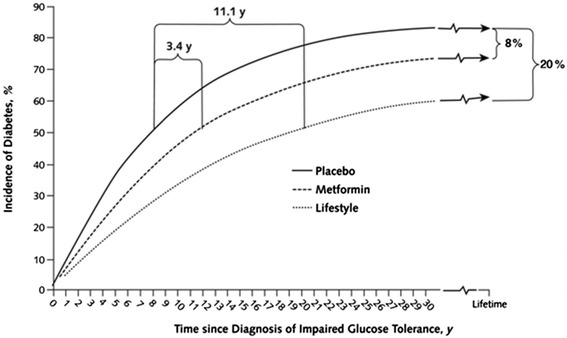
Simulated cumulative incidence of diabetes among adults with impaired glucose tolerance over a lifetime

The Fig. 1 illustrates the simulated lifetime cumulative incidence of type 2 diabetes by intervention group based on analysis of 3 years of data from the DPP [7]. With the placebo intervention, approximately 50 % of participants would develop diabetes within 7 years. In contrast, it would take approximately 18 years for 50 % of lifestyle participants to develop diabetes and 10 years for 50 % of metformin participants to develop diabetes. Thus, compared with the placebo intervention, the lifestyle intervention delayed the onset of diabetes by 11 years and metformin delayed the onset of diabetes by 3 years. Over a lifetime, 83 % of participants treated with the placebo intervention would develop diabetes, as compared to 63 % of those treated with the lifestyle intervention and 75 % of those treated with the metformin intervention. Thus, compared with the placebo intervention, the lifestyle intervention reduced the absolute risk of developing diabetes by 20 % and the metformin intervention reduced the risk of developing diabetes by 8 %. The number needed to treat (NNT), that is, the number of individuals that would need to be treated to prevent one additional case of diabetes over a lifetime, was 5 for the lifestyle intervention and 13 for the metformin intervention. The relative risk reductions were 24 % and 10 %, respectively.

Table 3 summarizes the simulated economic outcomes from the lifetime simulation. Compared with the placebo intervention, the lifestyle intervention cost $635 more over a lifetime and produced a gain of 0.57 QALY [7]. The cost per QALY (∆ cost/∆ QALY) was approximately $1100 [7]. Compared to the placebo intervention, the metformin intervention cost $3922 more over a lifetime and resulted 0.13 QALY-gained [7]. Thus, compared to the placebo intervention, the metformin intervention cost approximately $31,300 per QALY-gained [7].

**Table 3:** Simulated economic outcomes in the Diabetes Prevention Program intervention groups over a lifetime^a^

Outcome	Lifestyle intervention	Metformin intervention	Placebo Intervention
Lifetime intervention costs, $	9,718	8,801	2,907
Lifetime outcome costs, $	42,256	46,460	48,432
Total lifetime direct medical costs, $	51,974	55,261	51,339
Lifetime QALYs	10.89	10.45	10.32
∆ Cost vs. placebo, $	635	3,922	—
∆ QALY vs. placebo	0.57	0.13	—
∆ Cost / ∆ QALY, $	1,124	31,286	—

*QALY* quality-adjusted life-year

^a^Costs and QALYs discounted at 3 % per year

In sensitivity analyses, we found that compared to the placebo intervention, the lifestyle intervention was cost-saving in participants younger than 45 years of age and cost-effective in all age groups (Table 4). In contrast, the metformin intervention was cost-effective in the younger age groups but cost more than $100,000 per QALY-gained in participants 65 years of age and older. The lifestyle intervention was cost-effective in all age groups because it was effective in all age groups. The reduced cost-effectiveness of the metformin intervention in the older age groups was largely related to its reduced effectiveness in older participants.

**Table 4:** Simulated economic outcomes in the Diabetes Prevention Program intervention groups over a lifetime: Sensitivity analyses

Variable	Lifestyle Intervention vs. Placebo Intervention			Metformin Intervention vs. Placebo Intervention		
	∆ Cost, *$*	∆ QALY^a^	∆ Cost/∆ QALY, *$*	∆ Cost, *$*	∆ QALY^a^	∆ Cost/∆ QALY,*$*
Base-case analysis	635	0.57	1,124	3,922	0.13	31,286
Age 25–44 y	−395	0.63	Cost-saving	2,574	0.27	9,573
Age 45–54 y	489	0.63	781	4,024	0.13	30,013
Age 55–64 y	1,807	0.53	3,409	4,413	0.07	64,904
Age 65–74 y	2,617	0.39	6,646	4,119	0.02	173,593
Age ≥ 75 y	2,508	0.21	11,700	3,255	0.01	273,207
Reduced cost^b^	-3,696	0.57	Cost-saving	220	0.13	1,755
20 % reduced effectiveness	1,417	0.46	3,102	4,084	0.11	38,145
50 % reduced effectiveness	2,371	0.30	7,886	4,307	0.80	52,562
Reduced cost^b^ and 20 % reduced effectiveness	-2,181	0.41	Cost-saving	635	0.10	6,576
Reduced cost^b^ and 50 % reduced effectiveness	-348	0.23	Cost-saving	1,198	0.06	20,994

^a^ *QALY* quality-adjusted life-year

^b^Assumes that lifestyle intervention is implemented in a closed group of 10 patients and that metformin intervention is implemented with generic metformin

If the lifestyle intervention were implemented in a closed group of 10 patients and costs were reduced accordingly, and if the metformin intervention used generic metformin at 25 % the cost of Glucophage, the lifestyle intervention would be cost-saving relative to the placebo intervention and the metformin intervention would cost approximately $1800 per QALY-gained (Table 4). If future adherence were less than that observed in the DPP and the effectiveness of the lifestyle and metformin interventions were 20 % or even 50 % less than that observed in the DPP, the lifestyle intervention would cost $3100 to $7900 per QALY compared with the placebo intervention, and the metformin intervention would cost $38,000 to $52,600 per QALY (Table 4). If both the lifestyle and metformin interventions were implemented at lower costs, reflecting group lifestyle classes and generic metformin pricing, and effectiveness was reduced by 20 % or 50 % relative to that observed in the DPP, the lifestyle intervention would be cost-saving relative to the placebo intervention and the metformin intervention would cost approximately $6600 to $21,000 per QALY (Table 4).

### 10-year within DPP/DPPOS cost-effectiveness analysis

**Fig. 2:**
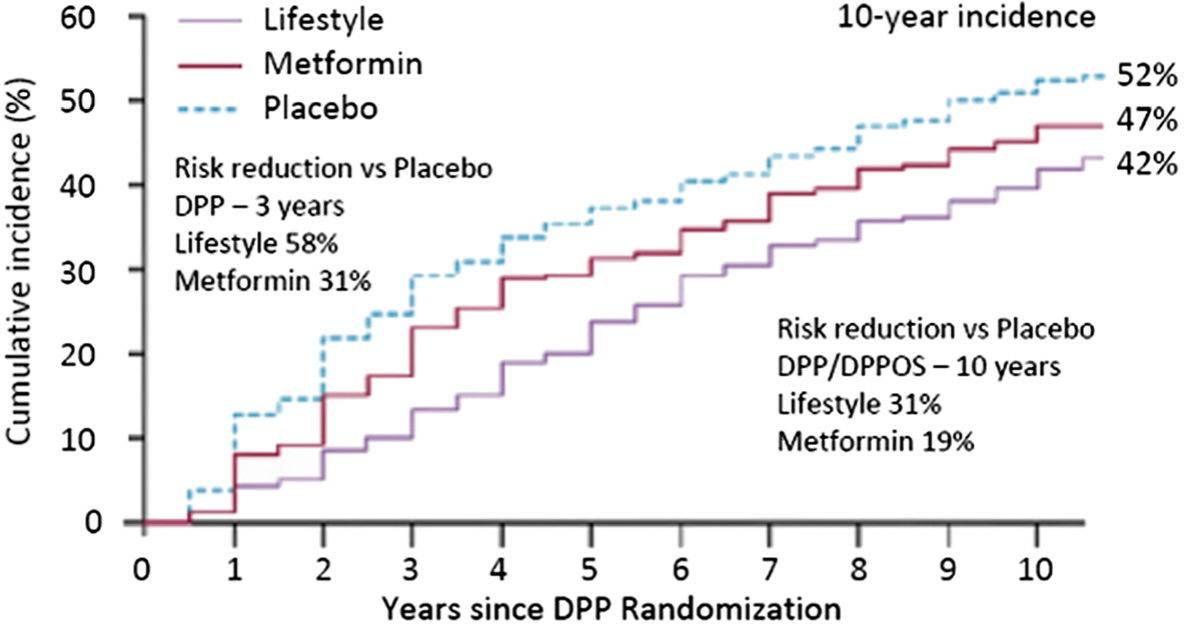
Observed cumulative incidence of diabetes among adults with impaired glucose tolerance over the combined 10-years of DPP/DPPOS

Figure 2 illustrates the effectiveness of the DPP/DPPOS interventions as assessed over 10 years of follow-up. After the conclusion of DPP, when all participants were offered a group lifestyle intervention, the relative difference in the effectiveness of the interventions decreased but the beneficial effects of the lifestyle and metformin interventions relative to the placebo intervention persisted.

Table 5 summarizes the differences in total costs and QALYs and the incremental cost-effectiveness ratios of the lifestyle and metformin interventions compared to placebo over the combined 10 years of DPP/DPPOS. The incremental cost-effectiveness ratio is also shown for DPP group lifestyle intervention compared to placebo. From the health system perspective and from the societal perspective, lifestyle cost more than placebo but was also more effective as assessed by QALYs-gained. From a health system perspective, with both costs and health outcomes discounted at 3 % per year, the cost of the lifestyle intervention compared to the placebo intervention was approximately $12,900 per QALY-gained. In contrast, from a health system and societal perspective, metformin had slightly lower costs and nearly the same outcome (as assessed by QALYs) as placebo. The DPP group lifestyle intervention cost approximately $1500 per QALY-gained from a health system perspective and $8400 per QALY-gained from a societal perspective after discounting.

**Table 5:** Differences in total costs and QALYs and incremental cost-effectiveness ratios for lifestyle and metformin vs placebo over 10 years

Differences in costs (∆ cost )	Lifestyle vs placebo	Metformin vs placebo	DPP group lifestyle vs placebo^a^
Health system perspective^b^			
Undiscounted	1,656	−251	$81
Discounted^b^	1,748	−205	$201
Societal perspective^d^			
Undiscounted	2,572	−3,644	$996
Discounted^b^	2,688	−3,021	$1,141
Differences in QALYs (∆ QALY)			
Undiscounted	0.15	0.01	0.15
Discounted^c^	0.14	0.01	0.14
Health system perspective^b^			
Undiscounted	10,759	Cost-saving	$528
Discounted^c^	12,878	Cost-saving	$1,478
Societal perspective^d^			
Undiscounted	16,699	Cost-saving	$6,468
Discounted	19,812	Cost-saving	$8,412

^a^Sensitivity analysis. Assumes that the core curriculum and follow-up visits were conducted as group session with ten participants during the 3 years of DPP

^b^Includes total direct medical costs

^c^Both costs and QALYs discounted at 3 %

^d^Includes direct medical costs, direct nonmedical costs including participant time, and indirect costs

## Conclusions

When a new treatment is cost-saving - that is, more effective and less costly than usual care, it should be widely adopted and used. Unfortunately, fewer than 1 in 5 new treatments in health and medicine is cost-saving compared to usual care [15]. Published cost-effectiveness ratios, that is the cost in dollars per QALY-gained for prevention and treatment range from less than $10,000 per QALY-gained to greater than $1 million per QALY-gained with most falling between $10,000 and $50,000 per QALY-gained [15]. While influenza immunization has been demonstrated to be cost-saving in the Medicare population, interventions such as mammography, antihypertensive treatment, and cholesterol treatment for secondary prevention of cardiovascular disease have been estimated to cost between $10,000 and $60,000 per QALY [16]. Widely implemented interventions such as dialysis for end-stage renal disease ($50,000 to $100,000 per QALY) and left ventricular assist devices ($500,000 to $1.4 million per QALY) are substantially more expensive [15, 16].

From the perspective of a health system or society, what is the value of delaying or preventing the development of type 2 diabetes? From a health system perspective, it delays or prevents the direct medical costs of diabetes including the costs of diabetes education and nutritional counseling, glucose monitoring, antihyperglycemic treatments, and surveillance and treatment of complications and comorbidities. From a societal perspective, diabetes prevention also reduces costs to the individual not reimbursed by the health system and time lost from work and usual activities. It also improves quality of life.

The direct medical costs of diabetes are enormous. The American Diabetes Association estimated that total per capita healthcare expenditures for people with diabetes are approximately $13,700 per year, of which, $7900 is attributable to diabetes [17]. This estimate likely overstates the costs of diabetes in DPP participants with diabetes since they were actively diagnosed, very early in their clinical course, and had few complications or comorbidities. In 2000, using data from a single managed care health plan, we estimated that the median, annual, direct medical cost of care for a man with diet-controlled type 2 diabetes with no microvascular, neuropathic, or cardiovascular risk factors or complications was approximately $1700 [13]. More recently, using data from approximately 7100 type 2 diabetic patients enrolled in 8 managed care health plans participating across the United States, we estimated that the mean, annual, per capita, direct medical costs of care would be approximately $2500 for a man with recent onset diabetes without complications or comorbidities [18]. These costs of uncomplicated type 2 diabetes are quite consistent with those observed during DPP/DPPOS. Compared to the substantial costs of diabetes, the costs of the lifestyle, metformin and DPP group lifestyle interventions were quite small.

During DPP and DPPOS, the costs of the lifestyle and metformin interventions were greater than the cost of the placebo intervention, but the cumulative, undiscounted, per capita costs of the lifestyle and metformin interventions were small in comparison to the cost of nonintervention-related medical care (medical care received outside the DPP/DPPOS). Indeed, within 3 years, the cumulative undiscounted costs of nonintervention-related medical care exceeded the 10-year cumulative direct medical costs of the lifestyle and metformin interventions. Within 10 years, the total, cumulative, undiscounted costs of the interventions and nonintervention-related medical care were only slightly higher for lifestyle than placebo and lower for metformin than placebo.

With respect to the cost-effectiveness of diabetes prevention, it is now clear that our use of a 3-year time horizon in our within-trial economic analysis [6] resulted in a higher cost per QALY-gained than the analyses which used a lifetime or a 10-year time horizon. With a three-year time horizon, treatment costs were higher and the benefits of the lifestyle and metformin interventions were less. The costs of both the lifestyle and metformin interventions were greatest in year 1, decreased substantially in years 2 and 3 and decreased further during years 4 through 10. In contrast, much of the benefit of the lifestyle and metformin interventions, as assessed by both cumulative, non-intervention-related direct medical costs and quality-of-life, occurred after three years of follow-up. The results highlight the importance of adopting a longer time horizon when assessing the impact of an intervention for a chronic disease.

The results of the 10-year within trial economic analysis of DPP/DPPOS support the results of our lifetime simulation. In the lifetime simulation, from the perspective of a health system, both the lifestyle intervention and the metformin intervention were cost-effective, and the results were robust to plausible changes in intervention cost and participant adherence. In the 10-year within trial economic analysis, lifestyle was cost-effective and metformin was marginally cost-saving or at least cost neutral compared to placebo.

There are at least two limitations to the 10-year within-trial analysis which might, in part, explain the difference between it and the results of our lifetime simulation. First, DPPOS was an observational follow-up of DPP, a randomized controlled clinical trial. It is likely that during DPPOS, when 57 % of placebo participants attended at least one group lifestyle intervention session, the placebo intervention was more effective than “usual care”. Thus, if real-world usual care were used for comparison, the difference in effectiveness between the lifestyle and placebo interventions might have been greater. Second, in our analysis of the DPP group lifestyle intervention, we assumed that lifestyle could be implemented in a group rather than individual format at one-third lower cost and achieve the same outcomes. Although group-implemented lifestyle interventions have been shown to be at least as effective as individual programs for weight loss, there has not been a direct comparison of individual and group lifestyle interventions for diabetes prevention.

Taken together, these analyses demonstrate that although more expensive, lifestyle intervention, when compared to placebo, is cost-effective, and generic metformin when compared to placebo is cost-effective or even cost-saving from a health system and societal perspective. If a DPP group lifestyle intervention could be delivered at 1/3 lower cost than the DPP lifestyle intervention and achieve the same outcomes, it might also be cost-saving compared to placebo.

The challenges associated with motivating a racially and ethnically diverse population to take up and maintain the weight loss and physical activity goals of the DPP over the long term should not be underestimated. Nevertheless, flexible interventions delivered by skilled lifestyle coaches that accommodate individual preferences and reflect local community and cultural contexts may achieve these goals [19–21]. In conclusion, these economic analyses should assist health plans and policy makers in comparing the benefit of diabetes prevention to other preventive and palliative interventions. The adoption of diabetes prevention programs by health plans and society will result in important health benefits and represents a good value for money.

## Additional file

Additional file 1:
**DPPOS Research Group Investigators.**

## Abbreviations

DPP: Diabetes Prevention Program
DPPOS: Diabetes Prevention Program Outcomes Study
