# Supplementary material for: The cost-effectiveness of diabetes prevention: results from the Diabetes Prevention Program and the Diabetes Prevention Program Outcomes Study
Source: Clin Diabetes Endocrinol. 2015 Sep 2;1:9. doi: 10.1186/s40842-015-0009-1 (PMC5471886; doi:10.1186/s40842-015-0009-1)
Supplement: Additional file 1: — DPPOS Research Group Investigators. [file 40842_2015_9_MOESM1_ESM.docx]

Pennington Biomedical Research Center (Baton Rouge, LA)

George A. Bray, MD*

Annie Chatellier, RN, CCRC**

Jennifer Arceneaux RN, BSN**

Amber Dragg RD, LDN**

Crystal Duncan, LPN

Frank L. Greenway, MD

Erma Levy, RD

Donna H. Ryan, MD

# University of Chicago (Chicago, IL)

David Ehrmann, MD*

Margaret J. Matulik, RN, BSN**

Kirsten Czech, MS

Catherine DeSandre, BA

***Jefferson Medical College* (Philadelphia, PA)**

Barry J. Goldstein, MD, PhD*

Kevin Furlong, DO*

Kellie A. Smith, RN, MSN**

Wendi Wildman, RN**

Constance Pepe, MS, RD

***University of Miami* (Miami, FL)**

Ronald B. Goldberg, MD*

Jeanette Calles, MSEd**

Juliet Ojito, RN**

Sumaya Castillo-Florez, MPH

Hermes J. Florez, MD, PhD

Anna Giannella, RD, MS

Olga Lara

Beth Veciana

***The University of Texas Health Science Center* (San Antonio, TX)**

Steven M. Haffner, MD, MPH*

Helen P. Hazuda, PhD*

Maria G. Montez, RN, MSHP, CDE**

Kathy Hattaway, RD, MS

Carlos Lorenzo, MD, PhD

Arlene Martinez, RN, BSN, CDE

Tatiana Walker, RD, MS, CDE

***University of Colorado* (Denver, CO)**

Richard F. Hamman, MD, DrPH*

Dana Dabelea, MD, PhD*

Lisa Testaverde, MS**

Denise Anderson, RN, BSN

Alexis Bouffard, MA, RN, BSN

Tonya Jenkins, RD, CDE

Dione Lenz, RN, BSN, CDE

Leigh Perreault, MD

David W. Price, MD

Sheila C. Steinke, MS

***Joslin Diabetes Center* (Boston, MA)**

Edward S. Horton, MD*

Catherine S. Poirier, RN, BSN**

Kati Swift, RN, BSN**

Enrique Caballero, MD

Barbara Fargnoli, RD

Ashley Guidi, BS

Mathew Guido, BA

Sharon D. Jackson, MS, RD, CDE

Lori Lambert, MS, RD, LD

Kathleen E. Lawton, RN

Sarah Ledbury, Med, RD

Jessica Sansoucy, BS

Jeanne Spellman, RD

***VA Puget Sound Health Care System and University of Washington* (Seattle, WA)**

Steven E. Kahn, MB, ChB*

Brenda K. Montgomery, RN, BSN, CDE**

Wilfred Fujimoto, MD

Robert H. Knopp, MD (deceased)

Edward W. Lipkin, MD

Ivy Morgan-Taggart

Anne Murillo, BS

Lonnese Taylor, RN, BS

April Thomas, RD, MPH, CDE

Elaine C. Tsai, MD, MPH

Dace Trence, MD

***University of Tennessee* (Memphis, TN)**

Abbas E. Kitabchi, PhD, MD, FACP*

Samuel Dagogo-Jack, MD, MSc, FRCP, FACP*

Mary E. Murphy, RN, MS, CDE, MBA**

Laura Taylor, RN, BSN, CDE**

Jennifer Dolgoff, RN, BSN**

Debra Clark, LPN

Uzoma Ibebuogu, MD

Helen Lambeth, RN, BSN

Harriet Ricks

Lily M.K. Rutledge, RN, BSN

Judith E. Soberman, MD

***Northwestern University’s Feinberg School of Medicine* (Chicago, IL)**

Mark E. Molitch, MD*

Boyd E. Metzger, MD*

Mariana K. Johnson, MS, RN**

Mimi M. Giles, MS, RD

Diane Larsen, BS

Samsam C. Pen, BA

***Massachusetts General Hospital* (Boston, MA)**

David M. Nathan, MD*

Mary Larkin, MSN*

Charles McKitrick, BSN**

Heather Turgeon, BSN**

Ellen Anderson, MS, RD

Laurie Bissett, MS, RD

Kristy Bondi, BS

Enrico Cagliero, MD

Kali D’Anna

Linda Delahanty, MS, RD

Jose C. Florez, MD, PhD

Valerie Goldman, MS, RD

Peter Lou, MD

Alexandra Poulos

Elyse Raymond, BS

Christine Stevens, RN

Beverly Tseng

***University of California-San Diego* (San Diego, CA)**

Elizabeth Barrett-Connor, MD*

Mary Lou Carrion-Petersen, RN, BSN**

Lauren N. Claravall, BS

Jonalle M. Dowden, BS

Javiva Horne, RD

Diana Leos, RN, BSN

Sundar Mudaliar, MD

Jean Smith, RN

Simona Szerdi Janisch, BS

Karen Vejvoda, RN, BSN, CDE, CCRC

***St. Luke’s-Roosevelt Hospital* (New York, NY)**

F. Xavier Pi-Sunyer, MD*

Jane E. Lee, MS**

Sandra T. Foo, MD

Susan Hagamen, MS, RN, CDE

***Indiana University* (Indianapolis, IN)**

David G. Marrero, PhD*

Kieren J. Mather, MD*

Susie M. Kelly, RN, CDE**

Paula Putenney, RN**

Marcia A. Jackson**

Gina McAtee**

Ronald T. Ackermann, MD

Carolyn M. Cantrell

Edwin S. Fineberg, MD

Angela Hadden (deceased)

Marion S. Kirkman, MD

Paris J. Roach, MD

***Medstar Health Research Institute* (Washington, DC)**

Robert E. Ratner, MD*

Vanita Aroda, MD*

Sue Shapiro, RN, BSN, CCRC**

Catherine Bavido-Arrage, MS, RD, LD

Lilia Leon

Peggy Gibbs

Gabriel Uwaifo, MD

Debra Wells-Thayer, NP, CDE

Renee Wiggins, RD

***University of Southern California/UCLA Research Center* (Alhambra, CA)**

Mohammed F. Saad, MD*

Karol Watson, MD*

Medhat Botrous, MD**

Sujata Jinagouda, MD**

Maria Budget

Claudia Conzues

Perpetua Magpuri

Kathy Ngo

Kathy Xapthalamous

***Washington University* (St. Louis, MO)**

Neil H. White, MD, CDE*

Angela L. Brown, MD*

Samia Das, MS, MBA, RD, LD**

Prajakta Khare-Ranade, MSc, RDN, LD**

Tamara Stich, RN, MSN, CDE**

Ana Santiago, RN

Cormarie Wernimont, RD, LD

***Johns Hopkins School of Medicine (Baltimore, MD)***

Christopher D. Saudek, MD* (deceased)

Sherita Hill Golden, MD, MHS, FAHA*

Tracy Whittington, BS**

Frederick L. Brancati, MD, MHS (deceased)

Jeanne M. Clark, MD

Alicia Greene

Dawn Jiggetts

Henry Mosley

John Reusing

Richard R. Rubin, PhD (deceased)

Shawne Stephens

Evonne Utsey

***University of New Mexico* (Albuquerque, NM)**

David S. Schade, MD*

Karwyn S. Adams, RN, MSN**

Claire Hemphill, RN, BSN**

Penny Hyde, RN, BSN**

Janene L. Canady, RN, CDE**

Kathleen Colleran, MD

Ysela Gonzales, RN, MSN

Doris A. Hernandez-McGinnis

Carolyn King, MEd

***Albert Einstein College of Medicine* (Bronx, NY)**

Jill Crandall, MD*

Janet O. Brown, RN, MPH, MSN**

Gilda Trandafirescu, MD**

Elsie Adorno, BS

Helena Duffy, MS, C-ANP

Angela Goldstein, FNP-C, NPP, CSW

Jennifer Lukin, BA

Helen Martinez, RN, MSN, FNP-C

Dorothy Pompi, BA

Harry Shamoon, MD

Jonathan Scheindlin, MD

Elizabeth A. Walker, RN, DNSc, CDE

Judith Wylie-Rosett, EdD, RD

***University of Pittsburgh* (Pittsburgh, PA)**

Trevor Orchard, MD*

Andrea Kriska, PhD*

Susan Jeffries, RN, MSN**

M. Kaye Kramer, BSN, MPH**

Marie Smith, RN, BSN**

Catherine Benchoff

Stephanie Guimond, BS

Jessica Pettigrew, CMA

Debra Rubinstein, MD

Linda Semler, MS, RD

Elizabeth Venditti, PhD

Valarie Weinzierl, MPH

***University of Hawaii* (Honolulu, HI)**

Richard F. Arakaki, MD*

Narleen K. Baker-Ladao, BS**

Mae K. Isonaga, RD, MPH**

Nina E. Bermudez, MS

Marjorie K. Mau, MD

***Southwest American Indian Centers* (Phoenix, AZ; Shiprock, NM; Zuni, NM)**

William C. Knowler, MD, DrPH*

Norman Cooeyate**

Alvera Enote**

Mary A. Hoskin, RD, MS**

Camille Natewa**

Carol A. Percy, RN, MS**

Kelly J. Acton, MD, MPH

Vickie L. Andre, RN, FNP

Roz Barber

Shandiin Begay, MPH

Brian C. Bucca, OD, FAAO

Sherron Cook

Jeff Curtis, MD

Charlotte Dodge

Matthew S. Doughty, MD

Justin Glass, MD

Martia Glass, MD

Robert L. Hanson, MD, MPH

Louise E. Ingraham, MS, RD, LN

Kathleen M. Kobus, RNC-ANP

Jonathan Krakoff, MD

Catherine Manus, LPN

Cherie McCabe

Sara Michaels, MD

Tina Morgan

Julie A. Nelson, RD

Christopher Piromalli, DO

Robert J. Roy

Sandra Sangster, RD

Miranda Smart

Darryl P. Tonemah, PhD

Rachel Williams, FNP

Charlton Wilson, MD

***George Washington University Biostatistics Center (DPP Coordinating Center*, Rockville, MD)**

Sarah Fowler, PhD*

Marinella Temprosa, PhD*

Michael D. Larsen, PhD*

Tina Brenneman**

Hanna Sherif, MS**

Sharon L. Edelstein, ScM**

Solome Abebe, MS

Julie Bamdad, MS

Melanie Barkalow

Joel Bethepu

Tsedenia Bezabeh

Nicole Butler

Jackie Callaghan

Caitlin E. Carter

Costas Christophi, PhD

Gregory M. Dwyer

Mary Foulkes, PhD

Yuping Gao

Robert Gooding

Adrienne Gottlieb

Nisha Grover

Heather Hoffman, PhD

Ashley N. Hogan

Kathleen Jablonski, PhD

Richard Katz, MD

Preethy Kolinjivadi, MS

John M. Lachin, ScD

Yong Ma, PhD

Qing Pan, PhD

Susan Reamer

Alla Sapozhnikova

***Lifestyle Resource Core***

Elizabeth M. Venditti, PhD*

Andrea M. Kriska, PhD

Linda Semler, MS, RD, LDN

Valerie Weinzierl, MPH

***Central Biochemistry Laboratory* (Seattle, WA)**

Santica Marcovina, PhD, ScD*

Greg Strylewicz, PhD**

John Albers, PhD

***NIH/NIDDK* (Bethesda, MD)**

Judith Fradkin, MD

Sanford Garfield, PhD

***Centers for Disease Control & Prevention* (Atlanta, GA)**

Edward Gregg, PhD

Ping Zhang, PhD

***University of Michigan* (Ann Arbor, MI)**

William H. Herman, MD, MPH

Michael Brändle, MD, MS

Morton B. Brown, PhD
